# Supplementary material for: Genetic characteristics of Jiaji Duck by whole genome re-sequencing
Source: PLoS One. 2020 Feb 12;15(2):e0228964. doi: 10.1371/journal.pone.0228964 (PMC7015413; doi:10.1371/journal.pone.0228964)
Supplement: Attachment — Submitted filename: The comments.docx [file pone.0228964.s007.docx]

In this study, the authors selected five duck populations with three individuals of each population to sequence the whole genome of each duck. Using these resequencing data, the authors explored out 1,447,932 JJ-specific SNPs which can be used as molecular markers, performed the genetic characteristics research of JJ and the genetic relationship between JJ and other duck breeds. Overall, the results of this paper are very interesting and it is an excellent paper. However, there are still some minor problems that should be modified.

1. The results of genetic relationship among these five duck populations should be mentioned in the abstract;

2. The tables presented in this paper should be changed to three-line tables;

3. please list the age and sex for the sequencing duck;

4. The description of sequencer is inconsistent in this paper;

5. The language should be improved by a native English speaker in order to eliminate grammatical and spelling errors and to conform to correct scientific English. I listed some of these errors below.

Line 30-33: “However, to date, the genetic characteristics and genetic relationship with other duck breeds has not been explored yet,” is not clear. The authors should refer to whose “genetic characteristics and genetic relationship with other duck breeds”;

Line 91: “other” should be changed to “the”;

Line 102: A comma should be placed behind “JJ and FF ducks”;

Line 251-252: The authors should delete the “on these genes”;

Line 275: should add a comma between FF and YD;

Line 298:should add blank space between the number and KB.
